# Atom-by-atom analysis of sintering dynamics and stability of Pt nanoparticle catalysts in chemical reactions

**DOI:** 10.1098/rsta.2019.0597

**Published:** 2020-10-26

**Authors:** Thomas E. Martin, Robert W. Mitchell, Edward D. Boyes, Pratibha L. Gai

**Affiliations:** 1 Department of Physics, University of York, York YO10 5DD, UK; 2 Department of Electronic Engineering, University of York, York YO10 5DD, UK; 3 Department of Chemistry, University of York, York YO10 5DD, UK; 4 York Nanocentre, University of York, York YO10 5DD, UK

**Keywords:** environmental scanning transmission electron microscopy, atom analysis, sintering dynamics, supported platinum systems, single-atom behaviour

## Abstract

Supported Pt nanoparticles are used extensively in chemical processes, including for fuel cells, fuels, pollution control and hydrogenation reactions. Atomic-level deactivation mechanisms play a critical role in the loss of performance. In this original research paper, we introduce real-time in-situ visualization and quantitative analysis of dynamic atom-by-atom sintering and stability of model Pt nanoparticles on a carbon support, under controlled chemical reaction conditions of temperature and continuously flowing gas. We use a novel environmental scanning transmission electron microscope with single-atom resolution, to understand the mechanisms. Our results track the areal density of dynamic single atoms on the support between nanoparticles and attached to them; both as migrating species in performance degradation and as potential new independent active species. We demonstrate that the decay of smaller nanoparticles is initiated by a local lack of single atoms; while a post decay increase in single-atom density suggests anchoring sites on the substrate before aggregation to larger particles. The analyses reveal a relationship between the density and mobility of single atoms, particle sizes and their nature in the immediate neighbourhood. The results are combined with practical catalysts important in technological processes. The findings illustrate the complex nature of sintering and deactivation. They are used to generate new fundamental insights into nanoparticle sintering dynamics at the single-atom level, important in the development of efficient supported nanoparticle systems for improved chemical processes and novel single-atom catalysis.

This article is part of a discussion meeting issue ‘Dynamic *in situ* microscopy relating structure and function’.

## 1. Introduction

Supported noble metal nanoparticles are used widely as heterogeneous catalysts for chemical processes in industry for the production of fuels, fuel cells, chemicals, healthcare and in pollution control, important for world economic development and sustainability. The size of a metal particle is a very important factor in its performance. There is increasing recognition that single atoms and sub-nanometre (nm) clusters can lead to different, and often better, activity and selectivity in chemical processes [1–3], based on dynamic correlations between increased catalytic activity and reduced surface atom coordination numbers under reaction conditions [4]. In heterogeneous catalysis employing supported metal nanoparticles, particle mean size growth through sintering can cause quantitative deactivation by the loss of surface area [5], and qualitatively by changes in particle form with size and environment. Nanoparticle deactivation due to sintering is generally explained in terms of two mechanisms: Ostwald ripening (OR), or particle migration and coalescence (PMC) ([5]; [6–10]). OR involves inter-particle transport and migration of mobile (atomic or molecular) species or adatoms, with larger particles generally growing at the expense of smaller ones, due to differences in the surface energy and adatom concentrations on the support. PMC involves the mobility and coalescence of particles, with growth in the mean size [11–16]. Thus far approaches to studying sintering of nanoparticles have been at the nanoparticle level, with numerous informative studies carried out to address the sintering mechanisms in terms of particle size distributions (PSD) [7–9,11,15]. This has been implemented via individual particle size tracking [6,9], by fitting theoretical models (including Monte Carlo simulations) to PSDs [8,17], using a mean-field approximation [5]. In the mean-field model, the growth and decay of nanoparticles is described in terms of interface-controlled and diffusion-controlled ripening models. They include a mean-field approximation where the concentration of atoms (or atomic species) on the support is constant beyond the screening distance from the edge of the particle, with this mean-field concentration governing the atom exchange for particles in the ripening process [5]. This approximation is elaborated in the literature [15,17]. Limitations of the mean-field model in understanding the OR mechanism are also highlighted [13,18]. Studies of PSDs in heterogeneous nanoparticle catalyst systems have indicated OR at low to moderate temperatures (up to several hundred °C) [9]. Recent studies on nanoparticles using aberration-corrected transmission electron microscopy (TEM) also report OR [11,16]. Based on PSDs, the early stages of rapid metal particle ripening in catalytic reactions and intermediate catalyst sintering are shown to lead to OR as the predominant sintering mechanism, with the process of sintering continuing even after thousands of hours of operation at higher temperatures.

Since sintering of nanoparticles can take place at the single-atom level, it is critical to have a better understanding of particle sintering mechanisms at that level to improve processes. However, obtaining insights into the mechanisms of particle sintering at the single-atom level has remained challenging because of the previous lack of reliable single-atom resolution under controlled reaction conditions. Single-atom images have long been reported using high-angle annular dark-field (HAADF) imaging in high vacuum scanning TEM (STEM) [19]. In HAADF imaging for atomic number (Z)-contrast, the image intensity is approximately proportional to Z^2^. Spherical aberration correctors have led to greatly improved probe resolution [20]. Methods to identify single atoms are reported in the literature [19,21] where single atoms appear as white dots in HAADF images and are quantified by intensity profiles and calibration of intensities [22]. MEMS heating holders, such as from DENSsolutions used here, provide much improved temperature control and stage stability.

Over the past two decades, there have been several key developments in electron microscopy techniques to image working catalysts, including nanoparticle catalysts, on the atomic scale. Atomic resolution-environmental electron microscopes have been designed and developed with controlled flowing gas environments surrounding the sample (referred to as gas-in-microscope design) at operating temperatures [23,24]. The gas-in-microscope developments have enabled atomic resolution studies of catalysts under environmental reaction conditions representative of technological environments. They include studies of dynamic single-atom interactions using single-atom resolution-environmental scanning transmission electron microscope (ESTEM) pioneered by Boyes and Gai [25,26] at the University of York. The ESTEM extends the earlier pioneering development of atomic resolution-ETEM of Boyes & Gai [23], which is used globally.

As the nature of the active reaction site of the metal nanoparticle, along with the processes by which the particle sinters and deactivates, have been shown to change both in magnitude and mechanism with the reaction environment [4,22,27–30], it is of fundamental importance to activate, react, observe and analyse nanoparticle systems in-situ, in real time, under controlled continuous gas flow reaction conditions at operating temperatures. Reacting supported single platinum atoms can now be resolved and analysed in controlled flowing reducing gas environments at operating temperatures in the ESTEM using HAADF imaging (ESTEM-HAADF) [3,31]. Single-atom dynamics in supported gold nanocatalysts in controlled redox environments at operating temperatures using the ESTEM have also been reported [27]. These studies are significant for the emerging field of single-atom catalysts (SAC) where one of the objectives is to maintain the stability of an active population of single atoms, or very small clusters, on a substrate. Previous studies reported investigations of static SAC carried out in a high vacuum EM at room temperature [2]. There are instructive reports on reaction processes over metal surfaces (especially over single-crystal metal surfaces) using scanning probe methods (SPM), which are summarized in a review [32]. In SPM methods tracking and analysis of dynamic single atoms between 3D nanoparticles, including any sub-surface contributions, in controlled continuously flowing gas environments at operating temperatures, are especially challenging. With the ESTEM, tracking and analysis of dynamic single atoms between three-dimensional nanoparticles present in catalysts and their interactions have been performed in controlled reaction environments in this study. Additional complexities have been uncovered in terms of discontinuous atom movements and local substrate pinning site interactions.

## 2. Experimental methods

### (a) Synthesis and in-situ analysis of Pt/C nanoparticles

Pt/C systems are of interest in chemical processes including for fuel cells, fuels and hydrogenation reactions and the production of chemicals more generally. We studied both model and technologically important practical (real-world) systems to understand the behaviour of the nanoparticles as a function of temperature in hydrogen gas environments, with single-atom resolution.

To prepare model Pt nanoparticles on carbon, a JEOL JFC2300HR sputter coater was used to deposit 0.2 nm of Pt onto the 5 nm thick C window supports on DENS SH30 Wildfire S3 MEMS chips with internal temperature measurements, purchased from DENSsolutions. Prior to the electron microscopy experiments, the samples were heated in high vacuum at 600°C to produce particles with radii in the range 0.5–1.5 nm. The particles were observed to be predominantly in [110] crystallographic orientations.

Practical (real-world) samples were prepared on 5 nm carbon-coated MEMS chips purchased from DENSsolutions. Aqueous H_2_PtCl_6_ solution (3 µl, 0.1 mM) was deposited onto the transparent window followed by calcination in H_2_ gas in a tubular furnace (40 ml/min H_2_, 200°C, 1°C/min, 1 h), forming polydisperse particles from about 1 nm to a few nanometre in diameter, with size ranges similar to those in the model system.

### (b) Dynamic in-situ ESTEM analysis

In-situ visualization and analysis of sintering of the nanoparticle system under controlled reaction environments were carried out at single-atom resolution, in real time, using a double-spherical aberration-corrected environmental (scanning) transmission electron microscope instrument (AC-E(S)TEM, JEOL 2200FS) designed and developed at York and operating at 200 kV [3,25,26,31,33,34]. ESTEM experiments were performed in flowing hydrogen gas as a function of temperature (T) and time (t). We first performed controlled dynamic sintering OR experiments on model Pt/C samples at 250°C in 3 Pa of H_2_ in the ESTEM. Several areas of the samples were examined to confirm the observations.

**Figure 1.**
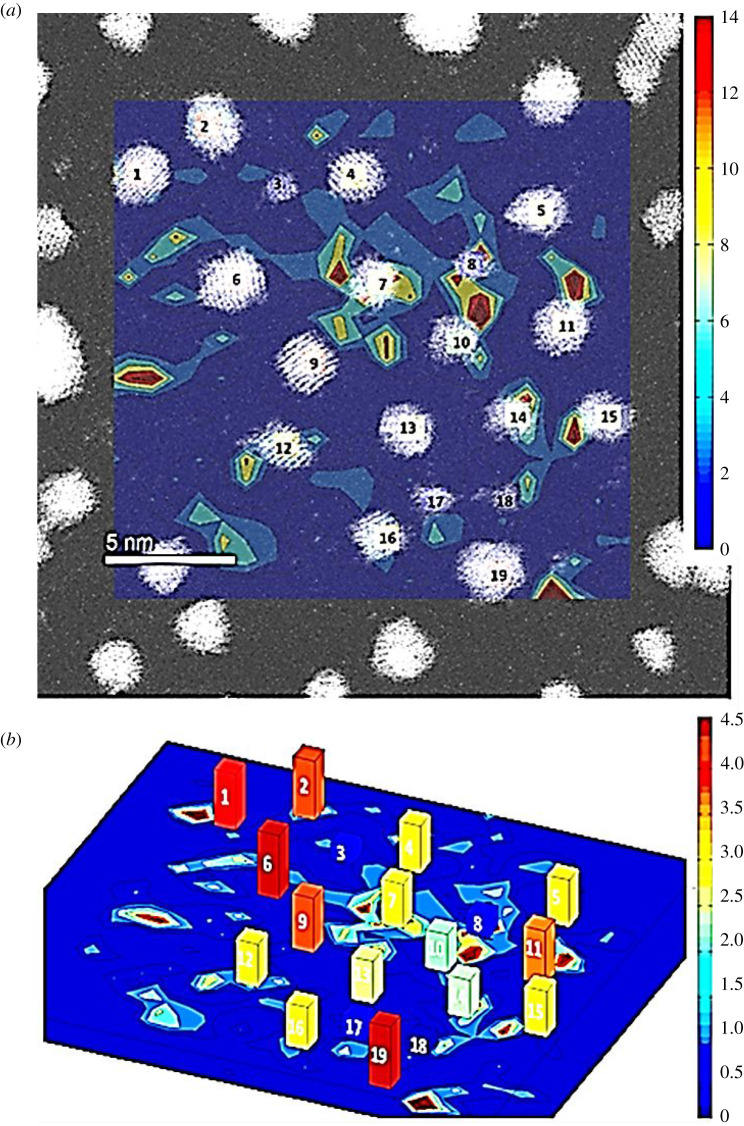
Single-atom heat map from *t* = 0 to *t* = 50 min, at which point no particles have become extinct. This is overlaid on an ESTEM-HAADF image at *t* = 0 (*a*). Heat map is rastered with a bin size of 0.76 × 0.76 nm. Particles 1–19 are labelled for reference purposes and the colour bar on the right applies to the single-atom count. (*b*) Heat map is the same, but particles in the overlaid image are replaced with bar plots to indicate the particle size. Colour bar on the right applies to the particle area in nm^2^. An averaging technique has been applied to smooth the single-atom data and form a contour plot. (Online version in colour.)

In the present work, a limited dose electron beam with careful calibration procedures and beam blanking were employed to avoid possible deleterious effects of the electron beam, with an estimated integrated dose rate exposure, averaged over the experiment, up to a few electrons per square angstrom per second [22,28]. Limited dose beam exposures were established empirically in each case. The data were checked with in-situ experiments under the same reaction conditions without the beam (blank calibration experiments by blanking the electron beam), with the beam switched on only to record the final reaction end-point. The particles here were only exposed to the beam during data set-up, interval examinations and final data acquisition. The images were generally 1024 × 1024, (using also other settings), with a pixel dwell time of 19.5 µs. Some images were also recorded with 2048 × 2048 pixels (bin) and 19.5 µs dwell time (figure 1). For the in-situ ESTEM studies, high-purity hydrogen gas (99.9995% from BOC, U.K.) was used with a pressure of 2–3 Pa. Gas pressures of a few Pa cover the sample surface with several thousands of monolayers per second of gas, fully sufficient to drive the chemistry [3,29]. The pressures are well above the surface science definition of high pressure region [35].

The application of ESTEM with consistent Z-contrast imaging of heavier single atoms allows detectability for a large observation area at modest (4Mx and up) magnifications. The ESTEM studies exploit differences in the higher atomic number (Z) contrast of metal single atoms, here on a thin (approx. 5 nm) low-Z carbon support. The TEM image contrast is dominated by diffraction and phase effects, which can make the visibility of supported single atoms and atomic species difficult [22,27]. The dynamic in-situ ESTEM experiments track the development, or degradation of nanoparticle nanostructures in real time, and the density of single atoms between three-dimensional nanoparticles present in working catalyst systems. Single atoms can operate both as migrating species in the nanostructure, influencing the long-term performance degradation of catalysts and as potential active species, but they were previously largely invisible to direct studies. The dependence of the particle size on single-atom density during sintering or OR can now begin to be studied on a rational basis using the ESTEM.

Previous work had observed the effects of OR through particle size changes as described in preceding sections, leading to an inferred understanding of the reaction mechanisms involved [7,9]. Other work has used scanning probe methods [6,32]. The focus of our work reported in this paper is to obtain an improved understanding of the dynamic relationship between the effect (particle size change) and the cause involving single-atom density and mobility, in a Pt/C nanoparticle system under controlled reaction conditions in a flowing gas environment at operating temperatures, using the ESTEM at the single-atom level. The findings presented here elucidate the complexity of OR and lead to a new level of detail in understanding sintering dynamics in OR, with implications for a more constructive nanostructural design basis for improved catalysts and processes.

## 3. Results and discussion

### (a) Analysis of experimental results

To define the interaction range of nanoparticles, and thus determine the local atomic concentration, a method known as the Voronoi construction has been reported [36]. This approach has been used by several authors to provide an enhanced understanding of sintering of Ag particles with Scanning Tunnelling Microscopy [6], Si with low-energy electron microscopy [36], and model platinum/silica with TEM [15]. The Voronoi approach is also followed here for Pt on carbon.

The application of the Voronoi approach [36,37] is that assuming a local model, variations in the single-atom density can be correlated to the particle size and decay. Local correlations, whereby single atoms are attributed to individual or groups of particles, have been explored here with the Voronoi construction. The region of influence of a particle consists of any point that is closer to that particle than to any other. Nearest neighbours are defined as particles that share a Voronoi boundary. The accuracy of the density measurement depends on single atoms detected being representative of the total population, limited by the detection efficiency, sample size and the migration rate. Understanding the limits to obtaining statistically significant single-atom counts is also important, as described in the electronic supplementary material, figure S1 (which illustrates the visibility of single atoms as a function of different acquisition parameters). In order to perform a statistical study on single atoms, mapping of constraints determining their detectability is required. Parameters such as temperature and reaction gas pressure affect the number and detectability of single atoms (as described in the electronic supplementary material). The electron beam scan rate dependence has been quantified using the Rose criterion, with the favourable analysis shown in the electronic supplementary material.

### (b) Quantifying single-atom density in dynamic reaction experiments

The key and complex task in relating the particle size to the single-atom density under controlled reaction conditions requires an adequate sampling to provide a representative overview of the dynamic processes. Constraints on the single-atom detectability, including the dependence on the migration speed of the atom versus the scan rate, are presented in the electronic supplementary material. Large datasets are required, with the number of single atoms detected much greater than the number of particles measured. If there are local fluctuations in the single-atom density, these can be used to spatially average the data and obtain improved statistics.

Here we present studies of both model systems and practical systems. The experimental findings and analyses are described in the following sections.

In the model system, we investigated a 23.5 nm × 23.5 nm area for 75 min at a sample rate of 1 acquisition per 5 min. The single-atom density was summed over time, *t* = 0–50 min. Eleven images were obtained during this period. This improves the statistics of the data and hence the precision of analyses. The data indicate that the ratio of single atoms to particles is increased by a factor of about 11 by time-averaging. In some areas of the sample, approximately 14 atoms per bin were observed and a diversity of single-atom distribution was detected in the sample. It is likely the rate of adatom migration is faster than the imaging rate (discussed in the electronic supplementary material), making it difficult to image and count the same adatoms each time. Nevertheless, from the dynamic observations presented here, some key conclusions emerge regarding the density and mobility of single atoms and the particle size change.

The diagram shown in figure 1 consists of 11 superimposed images from our dynamic ESTEM experiments, with each image containing a mean of 81 ± 19 single atoms. With a raster size of 0.76 × 0.76 nm, each bin should contain on average 0.08 atoms. Superimposed over 11 images this increases to 0.93 atoms per bin, according to the mean-field approximation [5]. This is considerably less than single-atom counts of up to 14 atoms per bin observed over areas of the sample in our experiments, illustrated in the colour bar (heat map) on the top right in figure 1. As summarized in the introductory section, the mean-field approximation [5] does not take into account different local particle environments (e.g. the size and number of surrounding particles) and assumes that the concentration of atoms on the support is constant. Our experimental data of spatially resolved local single-atom densities illustrate the behaviour of reacting atoms and indicate that the mean-field approximation is insufficient in this case. Examinations of several areas of the sample confirmed the observations. The detailed sampling is important as the data may be skewed by the presence of a large number of atoms in one region of a single image. This is examined in the electronic supplementary material, and with the methods used, found not to influence the results.

Higher single-atom densities are observed in the area containing particles 7, 8 and 10, with a much lower density found near 17 and 18. Particles 17 and 18 decay, while 7, 8 and 10 show area size fluctuations of 42%, but with no trend for decay. The observations lead to the significant conclusion that a higher density of single atoms implies higher exchange rates; as opposed to high net movement of mass between nanoparticles. Detachment of atoms can be understood by considering the following probabilities: emission, diffusion and reattachment; and that atoms detaching from nanoparticles are independent of the local single-atom density. A large total nearest-neighbour mass will increase the emission and reattachment probabilities. This illustrates the need to observe single-atom densities over the local area (nearest-neighbour regions) so that high or low local single-atom emitters can be explored.

**Figure 2.**
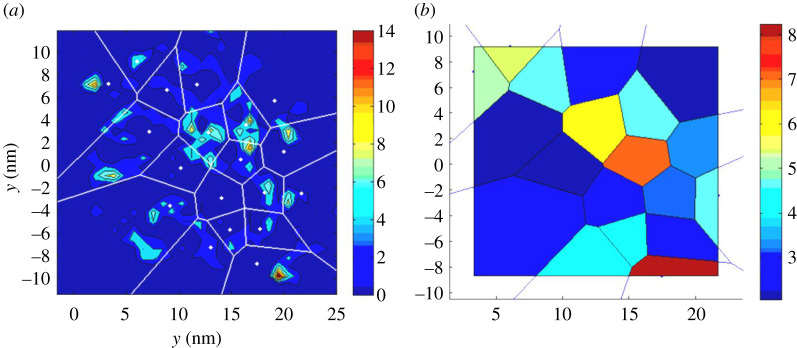
The local correlation model allows for spatial sampling of single-atom densities. (*a*) Heat map of single atoms from figure 1; with a Voronoi overlay (*b*) single-atom densities calculated using the Voronoi overlay instead of the raster. The colour bar (*a*) displays the number of single atoms per bin, (*b*) displays the single-atom density per nm^2^ in a Voronoi cell. (Online version in colour.)

The conversion between the raster map and the Voronoi cells can be seen in figure 2. When using the nearest-neighbour model, particles at the edge will be influenced by factors beyond the region of observation. Mitigation for this has been attempted by reducing the single-atom observation area to the inward facing sides of edge particles. This reduced sample area is illustrated in figure 2*b*. The solution assumes that single atoms observed on the outer facing sides of edge particles were not generated within the sample area (and vice versa for inward facing sides). The modification also disregards around 50% of the local area contributing to size changes of the edge particles. Ideally only non-edge particles should be examined. A Voronoi nearest-neighbour construction may also either average out or enhance trends in single cells. To corroborate the data, single Voronoi cell analysis has been performed and is presented in the electronic supplementary material.

### (c) Relating particle decay to single-atom density

Following the application of the Voronoi construction to the sample area, the decay of particles 17 and 18 are analysed by averaging single-atom numbers over a larger area (the nearest-neighbour Voronoi regions) as well as over time. This improves the statistics sufficiently to enable quantitative time-dependent single-atom density observations.

**Figure 3.**
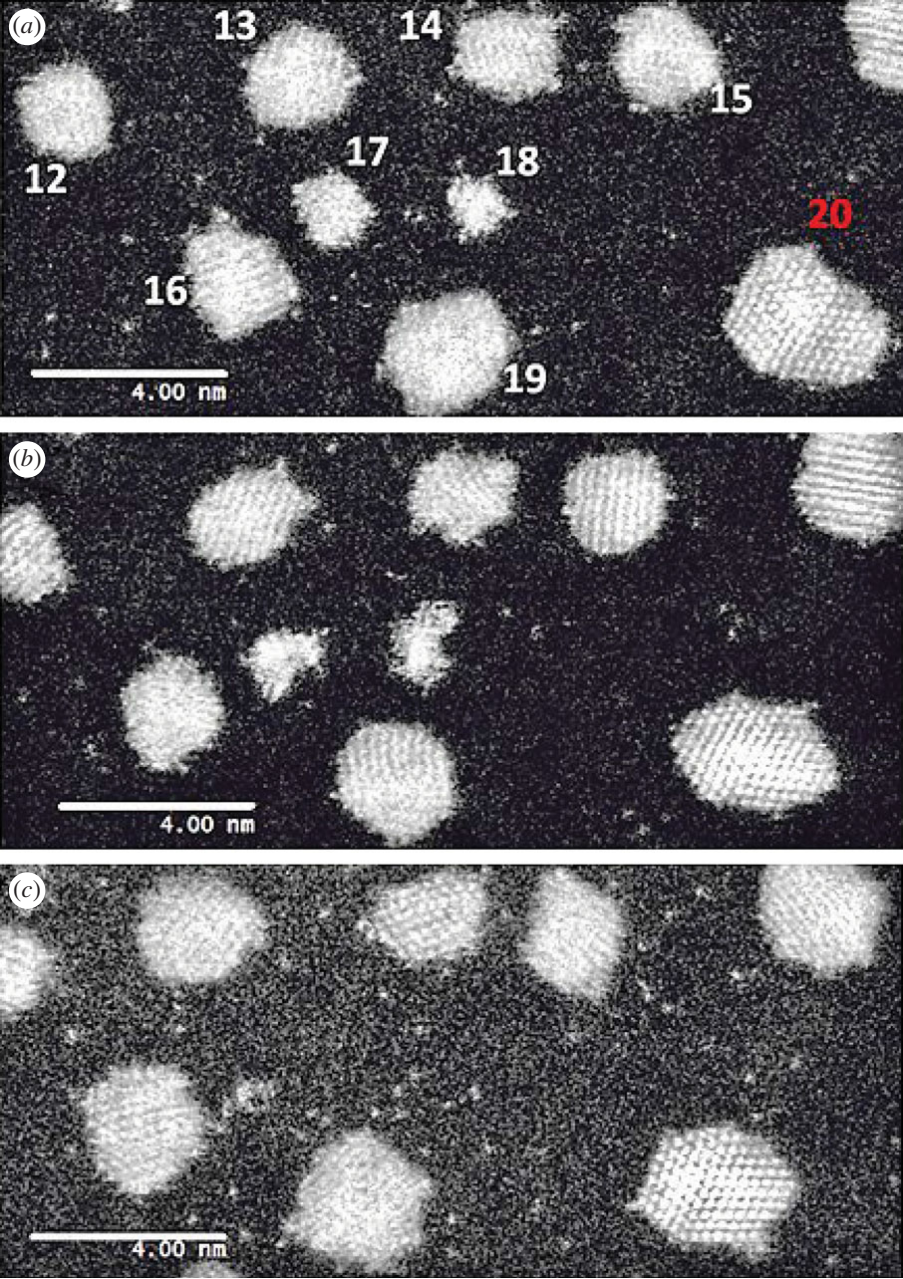
Dynamic in-situ ESTEM-HAADF observations in 3 Pa H_2_ at 250°C from the same local area of particles 17 and 18 and emissivity of single atoms. Images are recorded at time intervals of (*a*) *t* = 10 min, (*b*) *t* = 40 min and (*c*) *t* = 70 min. A decrease in the single-atom density is observed in (*b*), with an increased disorder of particles 17 and 18. Particle 20 (highlighted red) was not included in the Voronoi analysis, as it was not within the field of view of the microscope throughout the experiment. It is included here to illustrate the local environment of the particle 18. The dynamic observations reveal that as they are extinguished in (*c*) the local single-atom density increases, with details given in figure 4. Experimental brightness and contrast conditions were kept the same over the experiment as far as possible. (Online version in colour.)

Dynamic in-situ ESTEM-HAADF observations in 3 Pa hydrogen gas at 250°C from the same local area of particles are presented in figure 3.

Figure 3 illustrates that the single-atom density at (A) *t* = 10 min, is high, decreases considerably at (B) *t* = 40 min and then increases again after particles 17 and 18 have decayed at (C) *t* = 70 min.

**Figure 4.**
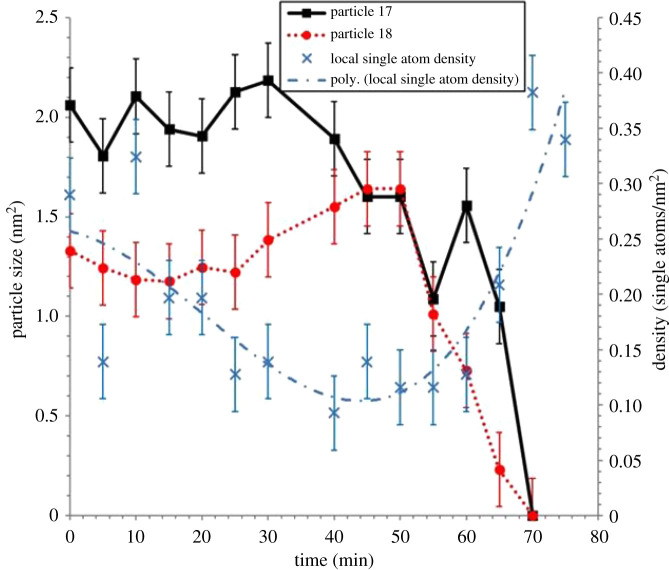
Plots of sizes of particles 17 and 18 during experimental time frame of 75 min. The dynamic sampling rate in the in-situ ESTEM studies is 1 acquisition per 5 min. The local single-atom density is calculated from Voronoi cells neighbouring particles 17 and 18, the cells included are: 12, 13, 14, 15, 16, 17, 18 and 19. A third-order polynomial has been fitted to the local single-atom density data, indicated by the dot-dash line. Errors in the particle sizes are calculated by measuring the change in the particle size. The error in the single-atom density is calculated by taking five successive images, counting the number of single atoms, then finding the corresponding single-atom density variations. (Online version in colour.)

A distinct correlation between the change in size of particles 17 and 18, and the density of single atoms in the nearest-neighbour Voronoi cells is shown in figure 4. Notably, our in-situ ESTEM observations have further revealed that the local single-atom density begins to decrease prior to particle size changes, indicating the decay of particles 17 and 18 is driven by the absence of single atoms to replace those emitted by the particles in a competitive dynamic process. The data also present evidence of interactions between particles 17 and 18. Once the concentration of single atoms decreases below the rate of emission from particle 17, the particle size begins to decrease at *t* = 30 min. This is accompanied by an increase in the size of particle 18, indicating that it is emitting at a lower rate. Eventually, when their sizes are approximately equal at *t* = 50 min, particle 18 also begins to decay, which corresponds with a spike in the size of the particle 17 at *t* = 60 min. Emission from both particles is a function of the relevant surface area with which atoms can interact to attach or detach more easily. This is the length and the nature of the external interface for interactions and interchange between material in or on the particle and that distributed across the support surface. In this case, the irregularity reduces the influence of the Gibbs–Thomson relationship [5] on emissivity. As a result, smaller particles can survive in a lower single-atom concentration due to their assumed lower emission. As described in the preceding sections, analyses from several areas of the sample have been carried out in our experiments, using imaging conditions with reduced dose beam currents, and parallel calibration procedures to confirm the data (Experimental Methods, electronic supplementary material figure S2). In the experiments, there is a measured increase in the single-atom density with time as particles 17 and 18 are extinguished. This supports the conclusion that throughout the experiment an adequate image contrast is maintained for the detection of Pt single atoms under the conditions employed.

Significantly, our dynamic observations have revealed that towards the end of the experiment at *t* = 60–70 min, the single-atom density increases again. Particles 17 and 18 decay to *r* = 0 at *t* = 70 min. Thus, a reduction in the single-atom density, once these net emitters have disappeared, would be expected. The increase in density over four observations (20 min) implies that the single Pt atoms are being anchored on the carbon support. Diffusion proceeds via a series of jumps between anchoring sites, with single atoms remaining stationary between hops. Hence the relative ease in imaging them. This is consistent with the conclusion reported in the literature [38], in which Au atoms remain stable on the C support for approximately 15 s before hopping approximately 2 nm each time. The anchoring of migratory single atoms on stabilized active sites for reactions is an area of increasing interest, e.g. W-species on grain boundaries of zirconia [33,39], and platinum on γ-Al_2_O_3_ [40].

### (d) Single-atom density as a function of particle size

The initial change in the single-atom density around particles 17 and 18 may be influenced by the changing particle structure of the nearest neighbours. Particles 3 and 8 are similarly irregular in external profile and with 17 and 18 make up the smallest four particles observed here. However, their local single-atom density appears to increase or stay constant over the experimental time frame (electronic supplementary material, figures S3 and S4). For understanding the relationship between particle size and single-atom density, knowledge of the particle surface, especially the nature of irregular (i.e. unfaceted, non-equilibrium high energy) external surfaces of the particle, is required. As stated in the preceding paragraphs, irregular particles are defined here as particles with irregular exterior surfaces in contact with the support where single atoms can be easily detached.

The decay of small nanoparticles with irregular external surfaces (17 and 18 illustrated in figure 4) indicates that they are inherently unstable. A rougher exterior of the particle is less stable with more detached atoms in the neighbourhood. When analysing a sample of several hundred nanoparticles, the particle size and the nature of particle exteriors are important contributors in identifying emission probabilities of disordered particles with irregular surfaces in contact with the support and larger well faceted particles with lower energy configurations.

**Figure 5.**
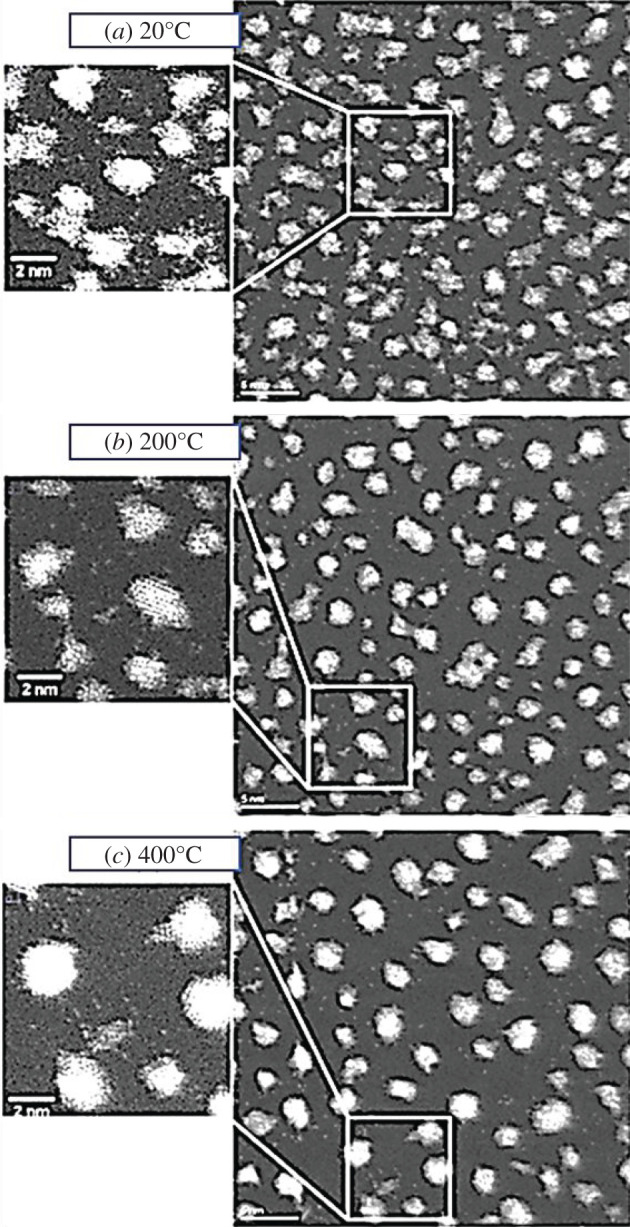
Real-time dynamic in-situ ESTEM-HAADF observations for correlating single-atom density with particle size by identifying nanoparticles with irregular (unfaceted) external surfaces and those with more faceted exteriors: (*a*) At 20°C, many particles with irregular surfaces and single atoms are present. As the temperature is increased to (*b*) 200°C and (*c*) 400°C, the number of irregular particles decreases and more faceting (more ordering) and fewer single atoms are observed. The scale bar in (*a–c*) is 5 nm. The scale bar in the magnified insets is 2 nm.

The results presented in figure 5 use the same experimental ESTEM conditions described in preceding sections to illustrate the nature of nanoparticle surfaces (with some areas magnified in the insets). Here Pt nanoparticles are synthesized without annealing, leading to a diversity of single-atom distributions, small irregular clusters of a few atoms and ‘rafts’, in the sample, as illustrated in figure 5*a*. Increasing the temperature is observed to lead to an increased faceting of more well-formed crystalline nanoparticles generally, as shown in figure 5*b,c*. The observations are consistent with the literature reports of enhanced faceting in irregular nanoparticles (sources of single atoms) under reduction reaction conditions [3,27,33] and non-crystalline particle transformations [41]. The primary parameters, including the reaction gas environment, operating temperature, the emission rate of single atoms, the diffusion speed and the extent of particle surface (exterior) faceting, affect the single-atom density.

We have used an approach here where the proportion of particles with irregular surfaces is altered in different experiments in order to relate the particle size and exterior to the single-atom density. The evidence that single-atom emission probabilities change with the particle size and exterior (shape) is obtained from a change in this relationship. This is achieved by increasing the temperature to reduce the number of non-equilibrium particles with irregular shapes. At low temperatures, our observations show that there are typically particles with irregular shapes (shown in figure 5*a*) but increasing the temperature to 400°C (figure 5*c*) increases the number of larger particles with equilibrium faceted shapes and fewer single atoms. A decrease in single atoms is attributed to the growth and faceting of nanoparticles. Our results reveal two distinct populations of nanoparticles in figure 5: there are many particles with irregular external surfaces at lower temperatures; and increased faceting and growth of the particles is observed at the higher temperature. The observations also reveal that some single atoms are still present at the higher temperature shown in (C). The emissivity of single atoms of clusters and small particles with irregular external surfaces is observed to be higher (e.g. particles 17 and 18 in figure 3), than that of well-established faceted particles, consistent with other reports (3,27).

Our findings elucidate that even after extended heating at 250°C single atoms, or very small clusters are retained. Figure 3, which is acquired after heating for 70 min at 250°C in hydrogen gas, is a notable example of this. These phenomena are not included in simulations of catalytic properties and nanoparticle sintering reported thus far (e.g. [7,9,10]). The new insights presented here also reveal that although many single atoms follow OR, some isolated single atoms continue after sintering, indicating the presence of pinning sites on the support in both OR and single-atom catalysis.

### (e) Dynamic sintering of practical Pt/C nanoparticle catalyst system

It is important to compare the behaviour of model catalysts with practical systems of technological interest. We studied dynamic sintering of the practical catalysts as a function of hydrogen gas and temperature in the ESTEM. Images were recorded at 4 Mx magnification, with 2048 × 2048 pixels, dwell times of 19 µs and 50 Hz line synchronization. Careful calibration procedures were followed throughout, as described in §2b. As described in the preceding sections, the electron beam was blanked during reactions and the beam was switched on only to record the final reaction end-point to minimize sample exposure and the data were compared with limited dose in-situ data. Regions with no previous exposure to the electron beam were also imaged periodically to probe any effect of the beam on particle sintering. The influences of the experimental conditions were investigated systematically by adjusting the beam on/off ratio, facilitated by the high stability of the MEMS stage employed, and gas pressure over the range 2–20 Pa. Single atoms were identified using the methods described in the preceding sections.

Suitable regions were identified for in-situ reaction experiments and unreacted samples were imaged initially in vacuum. Samples were subsequently heated under vacuum to remove any surface contamination and reacted in H_2_ gas to 300°C, then ramped in 100°C steps (30°C/min) over 80 min. Dynamic images were recorded in reaction environments at the operating temperature. (In some experiments after 80 min at 700°C the temperature was reduced to 300°C for imaging for comparison.) Experiments were also performed in which samples were heated to 350°C and 600°C for 1 h without beam exposure during reactions, with the beam switched on to record the reaction end-point.

**Figure 6.**
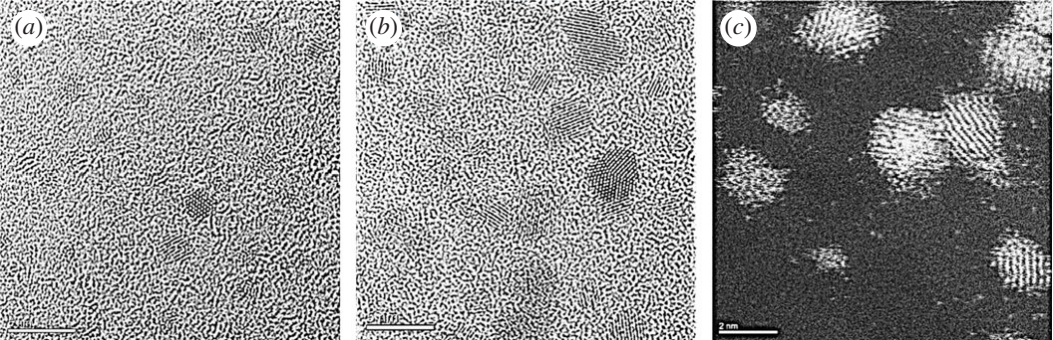
(*a*,*b*) Bright field TEM and (*c*) STEM-HAADF images of practical Pt nanoparticle catalysts recorded at 100°C under vacuum. Single atoms are clearly visible in the ESTEM-HAADF image in (*c*), but not in TEM images in (*a,b*). Scale bar = 5 nm in (*a,b*) and scale bar = 2 nm in (*c*).

The experimental data from the synthesis of the practical Pt/C system are presented in figure 6. The figure illustrates bright field (BF) TEM and ESTEM-HAADF images of Pt nanoparticles on carbon. Single atoms are not clearly visible in the BF TEM images (in (*a,b*)) due to diffraction and phase contrast effects, including of the 5 nm carbon support film, whereas they are reliably visible in the ESTEM-HAADF image shown in (*c*).

### (f) Single-atom dynamics in sintering and particle growth

**Figure 7.**
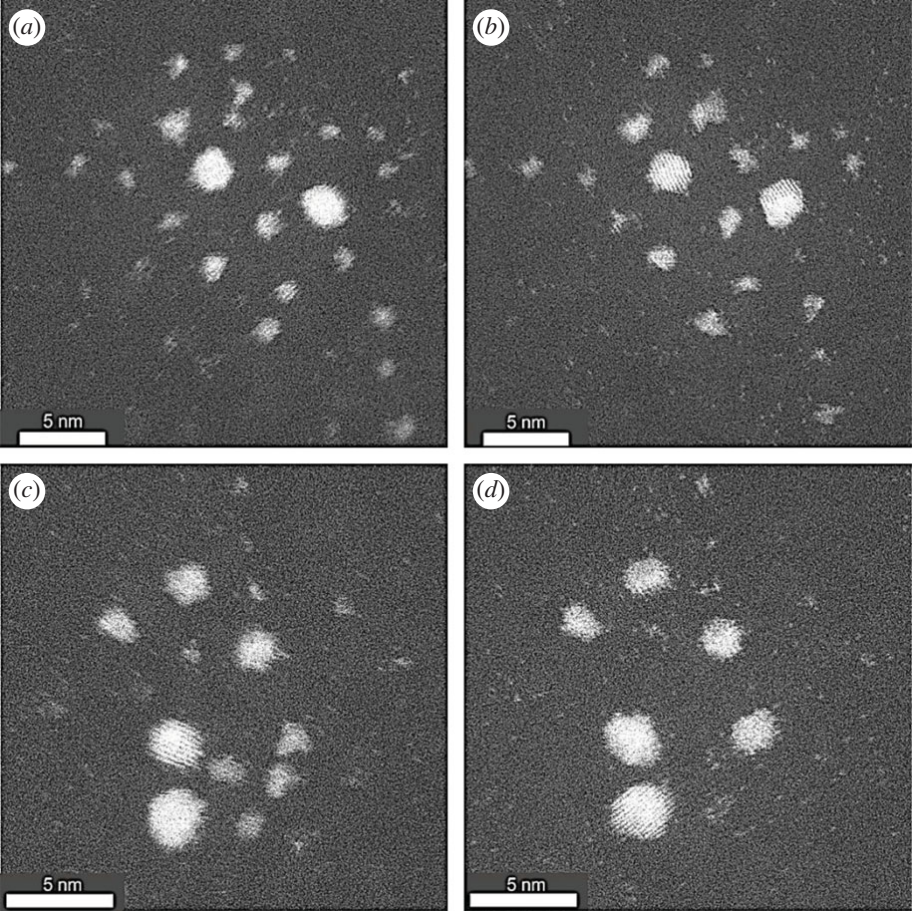
Real-time dynamic in-situ ESTEM-HAADF of Pt nanoparticle distributions of the same areas in the practical catalyst sample, imaged during reaction in H_2_ at operating temperatures in two areas: area 1 at top: (*a*): 350°C for 1 h (at left) and (*b*) 600°C for 1 h (right); and from area 2 at bottom: (*c,d*) 350°C for 1 h and 600°C for 1 h, respectively; single atoms are still clearly visible at the high operating temperature of 600°C. The higher temperatures have caused some smaller irregular particles and clusters in the images to be diminished or lost altogether, and adjacent larger ones to grow or be reformed. Scale bar = 5 nm.

We studied the growth of the supported practical platinum nanoparticles as a function of time, and temperature in an environment of hydrogen gas. Figure 7 shows the Pt nanoparticles in hydrogen gas at 350°C and 600°C. Importantly, single atoms are still visible at the higher temperature of 600°C under the reaction conditions.

**Figure 8.**
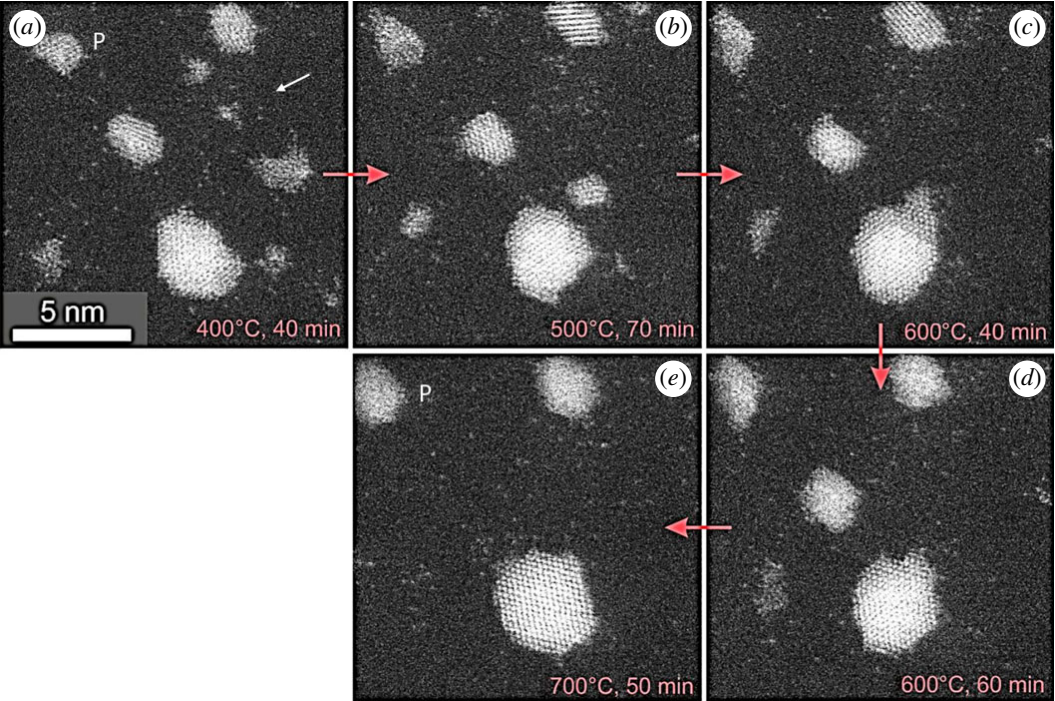
Dynamic in-situ ESTEM-HAADF image series as a function of temperature and time, from the same area of the sample (near P), showing faceting, partial outer layers and rearrangement of platinum nanoparticles. (*a*) 400°C, 40 min (*b*) 500°C, 70 min; (*c*) 600°C, 40 min (*d*) 600°C, 60 min; (*e*) 700°C, 50 min. Pt single atoms and clusters are visible at the elevated temperatures.

To evaluate the mobility of single-atom species and rearrangement of nanoparticles as a function of temperature and gas environment, regions of fresh samples were imaged at each temperature during a series of reactions. The migration of single atoms and the growth of the nanoparticles were studied as a function of temperature and time, both in vacuum and under hydrogen gas environments. These are illustrated in the following figures. Figure 8 shows the rearrangement and increased faceting and texturing of a Pt nanoparticle heated to 700°C under vacuum.

**Figure 9.**
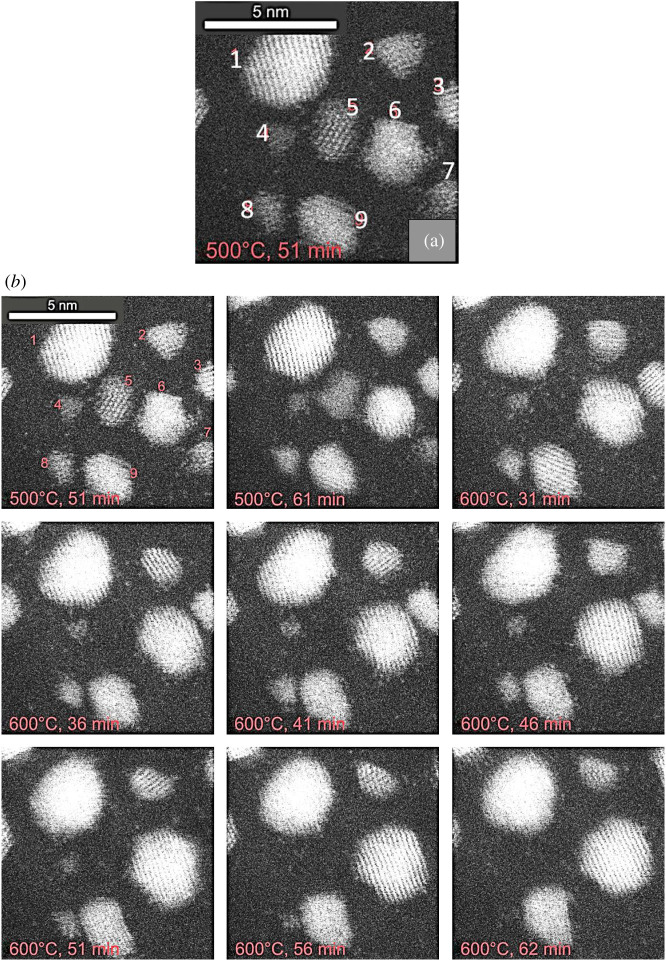
Real-time dynamic in-situ ESTEM-HAADF images of a reaction sequence in H_2_ gas as a function of temperature and time, from the same region of practical Pt/C sample, illustrating single-atom dynamics and the effect on the size and stability of the Pt nanoparticles on the support. Nine particles are identified in the first image shown at the top in (*a*) and are monitored in the following sequence. (*b*) Images from left to right: top row: at 500°C after 36 min, 41 min and 46 min; middle row at 600°C, after 36 min, 41 min and 46 min; and bottom row at 600°C after 51 min, 56 min and 62 min. Scale bar is 5 nm and applies to all the images. (Online version in colour.)

A dynamic image sequence in hydrogen gas, in real time, is shown in figure 9. Nine particles are identified in the first image shown in (*a*). Through the sequence (shown in (*b*)), small irregular particles 4 and 8 are observed to shrink and eventually decay after 56 and 62 min, respectively, at 600°C. The migration of single atoms is observed between the nanoparticles. The process is slow enough to observe on this timescale. (See earlier section on the details of this conclusion.) The observations indicate that the decay of smaller particles is associated with the emission of single atoms and their reattachment elsewhere; overall resulting in the growth of larger particles.

After 31 min at 600°C, particle 6 has grown at the expense of particles 5 and 7. In the images recorded after 46 and 51 min, the particles 3 and 6 coalesce, and in subsequent images rearrangement of the particle is observed. Thus, the sequence indicates Ostwald Ripening and decay of small particles/coalescence occurring at 600°C. Dynamic studies of single-atom interactions in supported gold nanoparticles in redox cycles have been reported [27].

The progression of the particle size in the reactive hydrogen gas environment as a function of temperature in figure 9 is shown in table 1.

**Table 1.** Particle size evolution for images in figure 9.

	particle area nm^−2^			
particle number	500°c, 51 min	600°C, 31 min	600°C, 56 min	600°C, 62 min
1	8.2	8.8	8.9	9.2
2	1.8	2.0	1.5	1.5
6	4.2	6.5	7.9	8.2
9	3.0	3.6	3.9	4.0

### (g) Single-atom distribution

**Figure 10.**
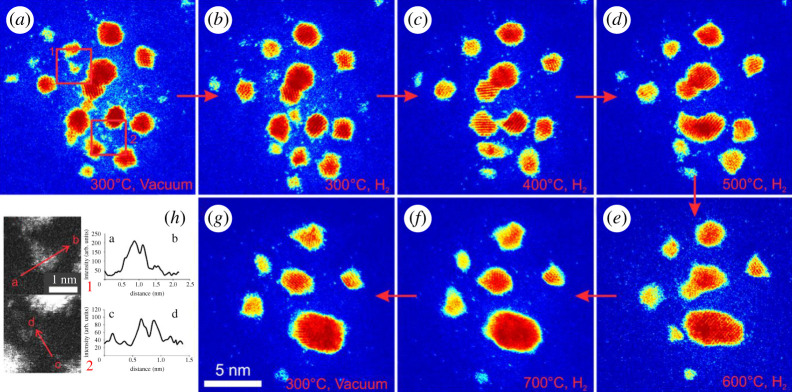
(*a–f*): Real-time dynamic in-situ ESTEM-HAADF images of practical Pt nanoparticles on carbon, in hydrogen gas as a function of operating temperature, revealing single-atom dynamics. The observations show the migration of single atoms and the effect on the nanoparticles during the reaction in hydrogen gas, as a function of the reaction temperature (where the temperature is increased by 100°C every 80 min): (*b*) 300°C; (*c*) 400°C; (*d*) 500°C; (*e*) 600°C; (*f*) 700°C. ((*g*) Dynamic image in vacuum at 300°C after the reaction is shown for comparison); (*h*) line intensity profiles of regions 1 and 2 in (*a*) indicate the presence of Pt single atoms and clusters. (Online version in colour.)

Single-atom species are observed to migrate on the support between the nanoparticles during reduction in hydrogen gas. The migration of single atoms and its effects on the particle dynamics, stability and size during the hydrogen reaction are illustrated in figure 10 as a function of temperature and time. The figure elucidates the migration of single atoms recorded from the same region of the sample: figure 10*a–d* illustrates the emission of single atoms from irregular (unfaceted) smaller particles and decay of the smaller particles: from left to right: (*a*) image recorded in vacuum at 300°C; (*b*) in hydrogen at 300°C; (*c*) in hydrogen at 400°C; in hydrogen at 500°C. Bottom row: from right to left (*e*) in hydrogen at 600°C and (*f*) in hydrogen at 700°C. (An image recorded in vacuum at 700°C is also shown in (*g*) for comparison.) Figure 10*h* shows line intensity profiles of regions in (*a*), displaying pronounced intensity from single-atom regions in comparison to the background intensity. Significantly, the in-situ dynamic observations reveal that single atoms are observed at elevated temperatures of 700°C in H_2_ gas environment. They further show that atoms can move to nanoparticles and lead to the growth of the particles. Within the error margins, the data are consistent with the conservation of mass for nanoparticles.

**Figure 11.**
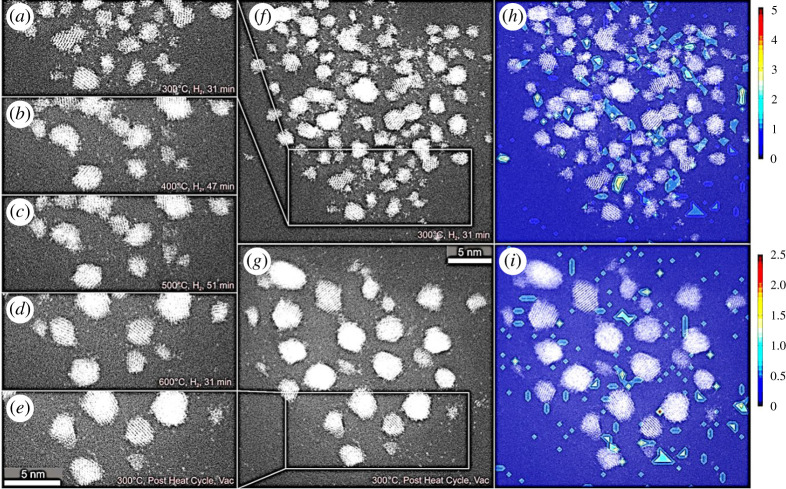
Dynamic in-situ ESTEM-HAADF image series showing the migration of single atoms and changes in platinum nanoparticles during reaction in hydrogen gas. Region imaged at 5 min intervals from 30 to 70 min. (*a–d*) are recorded in hydrogen at 300, 400, 500 and 600°C, respectively. The particles grow and facet at increasing temperature. (*e*) Image in vacuum at 300°C (after the gas reaction at 600°C) recorded for comparison. Panels (*f*) and (*g*) show enlarged regions of (*a*) and (*e*), respectively. Panels (*h*) and (*i*) show single-atom density heat maps for (*f*) and (*g*) and show the variation and diversity of single-atom distribution in the sample: (the top map (for *h*) shows from 0 to 5. In the bottom map (for *i*), from 0 to 2.5, showing faceted particles and a decrease in single atoms). Colours correspond to density of atoms in 50 × 50 pixel bins. (Online version in colour.)

Dynamic image sequences and the corresponding heat maps showing the evolution of platinum nanoparticles during reaction in hydrogen gas as a function of operating temperature of up to 600°C are illustrated in figure 11. The region was imaged at 5 min intervals from 30 to 70 min. (*a*), (*b*), (*c*) and (*d*) are recorded in hydrogen at 300, 400, 500 and 600°C, respectively. The observations show the number of faceted particles increase with temperature and a decrease in single atoms. Panel (*e*) is the image in vacuum at 300°C (after the gas reaction at 600°C), recorded for comparison. Panels (*a*) and (*e*) are magnified in (*f*) and (*g*) and show the particle evolution as a function of temperature; (*h*) and (*i*) show single-atom density heat maps for (*f*) and (*g*), respectively. Panels (*h*) and (*i*) show single-atom density heat maps for (*f*) and (*g*). They show the variation and diversity of single-atom distribution in the sample.

In figure 11, particles grow from 1.29 ± 0.35 nm (76 particles) in (*f*) to 2.00 ± 0.66 nm (26 particles) in figure 11 (*d* and also *g*) after reduction cycles in hydrogen gas. The data are again consistent with the conservation of mass for the particles within experimental error margins. The observations in both figures 10 and 11 show the same trend in the single-atom density, with a decrease during and after the reaction treatments. The decrease in the single-atom concentration and adatom emission are attributed to the growth and increased faceting of particles (as observed in figures 10 and 11). This is supported by the single-atom heat maps shown in (*h*) and (*i*) of figure 11. Our dynamic observations reveal that in general the well-established faceted (ordered) particles which show ordered crystalline lattice have fewer single atoms in close proximity than the more raft-like irregular (disordered) particles.

### (h) Discussion of single atom dynamics

The findings of practical samples are consistent with those in the model system. As described in preceding sections, irregular (or disordered) particles are defined as those with irregular exterior surfaces in contact with the support where single atoms can be detached, indicating that the particle thickness in three dimensions (or thicker particles) is not relevant.

The results can be rationalized by understanding that the mobility, and conversely site stability, of an individual atom on an exterior site is controlled by the activation energy for motion. This depends in part on the local bonding to adjacent Pt atoms, if present, and with the support. It follows that a particle edge which is ragged (irregular) is also less stable, because the exterior atom has fewer nearest neighbours to support it. This situation will be more prevalent in a single-atom thick raft-like particle, perhaps itself with less well-formed bonding and structure internally, than for a site on a well-established three-dimensional network; even if that comprises only a second layer of atoms, because that will still provide additional nearest-neighbour bonding. There will also be circumstances in which the smaller number of nearest neighbours can be expected to also increase the reactivity of the site. The findings on the nanostructure in the real catalyst are consistent with those in the model system. In the latter, the association of higher local atom density with decaying particles (figures 3 and 4) are excellent examples, also seen in figures 9–11, but this is an aspect of the study which needs to be followed up in more detail elsewhere.

Currently tracking of reacting atoms is a great challenge. Since atoms are identical it is difficult to identify the atom as it moves, except by being able to image at a frame rate frequency fast enough to identify the atom in the new position by the unique absence in an adjacent site in a prior image. This is a limitation of current methods, where the necessary high quality requires less than 1 Hz frame rate and minimally invasive imaging conditions are not fast enough. Regarding where the atoms go, by making the reasonable assumption of constant mass under reducing reaction conditions, the data are consistent with classical OR theory in which on average smaller particles progressively lose atoms which are aggregated to larger ones via migration over the support surface, as observed in our dynamic ESTEM experiments (e.g. in figures 3 and 10). The aggregation of single atoms to large particles is observed in an exact zone axis orientation but their source site cannot be individually identified with them. The results are therefore on a statistical basis of the overall effect and mechanisms of deactivation through sintering.

The dynamic in-situ ESTEM observations of the practical Pt/C system have revealed the migration of single Pt atoms between Pt nanoparticles on the carbon support, with a population of single atoms existing among more established nanoparticles, and in continuous interchange with them over a considerable length scale, in a competitive process. Again, as observed for the model systems, our findings show that single atoms continue to exist in hydrogen gas at much higher temperatures, such as 700°C for Pt, than previously anticipated. The practical Pt/C has been synthesized through a fully scalable route. The observations of single-atom dynamics in sintering are relevant to chemical processes employing Pt/C systems in industrial catalysis and open up opportunities for dynamic single-atom studies in other complex practical catalysts. The in-situ observations reveal that there is a continuing population of single atoms with important implications on catalyst reactivity, as well as providing the atom-by-atom deactivation mechanism.

## 4. Conclusion

Quantitative in-situ ESTEM observations of nanoparticle sintering have been carried out for the first time at the single-atom level to detect and study sintering dynamics of single Pt atoms on carbon supports in controlled environments of continuously flowing H_2_ gas at operating temperatures. The findings have revealed an important relationship between the single-atom density and changes in the particle size. The dynamic observations elucidate the relationship of single-atom density with a specific particle, or its nearest neighbours and that the particle decay is initiated by a lack of local single atoms. A post decay increase in the single-atom density indicates that anchoring sites exist on the support with the diffusion of single atoms governing OR. The findings reveal the complex nature of sintering and add a new level of detail to understanding the OR mechanism, albeit in the constrained two-dimensional free surface geometry of this application. The results have important implications for understanding the site stability of reacting single atoms in working nanoparticle catalyst systems, and on their role in particle coarsening and deactivation. The new results from the single atom resolution-ESTEM capability recently introduced by Boyes and Gai [25,26,33] to reliably detect single Pt atoms on the support or attached to nanoparticle surfaces under controlled reaction conditions inform future nanostructural developments for more efficient supported metal nanoparticles for hydrogenation and related chemical processes. The density of single atoms between the nanoparticles is observed to decrease after reactions in a hydrogen environment at operating temperatures. The dynamic in-situ ESTEM observations under controlled reaction conditions provide an improved understanding of single-atom dynamics and the effect on particle sintering and size during reactions, important in the optimization of supported nanoparticle catalysts.

## Supplementary Material

Atom-by-atom analysis of sintering dynamics and stability of Pt nanoparticle catalysts in chemical reactions using in-situ environmental scanning transmission electron microscopy

## Data accessibility

This article has additional data in the electronic supplementary material.
