# Supplementary material for: Atom-by-atom analysis of sintering dynamics and stability of Pt nanoparticle catalysts in chemical reactions
Source: Philos Trans A Math Phys Eng Sci. 2020 Oct 26;378(2186):20190597. doi: 10.1098/rsta.2019.0597 (PMC7661282; doi:10.1098/rsta.2019.0597)
Supplement: Atom-by-atom analysis of sintering dynamics and stability of Pt nanoparticle catalysts in chemical reactions using in-situ environmental scanning transmission electron microscopy [file rsta20190597supp1.pdf]

Supporting Information

Atom-by-atom analysis of sintering dynamics and stability of Pt nanoparticle catalysts in chemical reactions

Thomas E. Martin [1, 4], Robert W. Mitchell [1, 4], Edward D. Boyes [1, 2, 4 *] and Pratibha L. Gai [1, 3, 4 *]

[1] Department of Physics, [2] Department of Electronic Engineering, [3] Department of Chemistry, [4] York Nanocentre, University of York, York, YO10 5DD, U.K.

Contents

1. **Single Atom Detectability**
2. **Voronoi Averaging Effects**
3. **Acknowledgements**
4. **References**

Corresponding Authors

* Email: ed.boyes@york.ac.uk

* Email: pratibha.gai@york.ac.uk

1. Single Atom Detectability

In order to perform a statistical study on single atoms, the constraints determining their detectability in ESTEM must be mapped out. To begin with, the reaction conditions of temperature and gas environments will be expected to alter the number and detectability of single atoms. Temperature is held constant at 20˚C, whilst single atom resolution in H_2 has been previously shown to be at the sub-Angstrom level.

The next constraint on single atom detectability, as suggested in [1], is the dependence on the migration speed of the atom versus the scan rate of the electron beam. For a scan speed much slower than the migration speed, the atom will only be present under the electron beam for a fraction of the dwell time. This scenario leads to the atom not being imaged at all, imaged with a very low SNR (signal to noise ratio) and/or being imaged multiple times. If the scan speed and migration speed are approximately equal, then a smearing of the atom intensity across several pixels may occur. There are two frequencies at which this can transpire, one for the horizontal scan speed and another for the vertical scan speed. The detectability of the atom then also becomes dependent on its direction, as well as its speed.

The scan rate must therefore be much faster than the single atom migration speed for a representative population of single atoms to be detected. As the migration speed may be affected by temperature and the support, the appropriate sampling rate must be determined on a case by case basis.

In addition to these detectability constraints, there is also the question of obtaining a statistically significant sample. For relating particles to single atoms, the requirement is that many single atoms

are detected per particle. The number of particles sampled must also be as large as possible. The challenge is therefore to use a low magnification, whilst maintaining single atom resolution by altering the pixel size and dwell time. The limiting factor in the case of Pt/C is the variation in the support height perpendicular to the electron beam. For magnifications <4MX, the electronic focus must be adjusted during image acquisition to account for height variations. By using a 4MX magnification, this issue is avoided and a sufficient sample size (approximately 200 single atoms and 100 particles) is obtained with this particle size distribution.

The next constraint is on sample drift. Generally, if the acquisition time is much larger than 2 minutes (clearly this value will fluctuate with microscope conditions), the position of features are perturbed relative to each other. The maximum acquisition time used is therefore 81.8s, but the proportion of this devoted to either number of pixels or dwell time can be chosen. To quantify this, pixel size and dwell time are independently varied at 4MX magnification. The signal from 30 single atoms is sampled along with 30 regions of the same size containing only the C-film. The region sampled is kept at 0.3x0.3nm (although the number of pixels varies) and is therefore comparable in diameter to a Pt atom [2]. To enable contrast measurements, the mean grey value of the sample region is calculated.

Here the contrast is defined as the signal difference between a single atom (S_A) and the C-film (S_B). The contrast to noise ratio can then be defined as:

$$CNR = \frac{S_A - S_B}{\sigma_0} \tag{1}$$

In an image a single atom occupies n pixels, each with a signal of S_A with independent additive variation between the individual measurements of standard deviation σ_0. The effective local SNR will be improved by the number of pixels making up a single atom. The CNR is modified by $\sqrt{n}$, thus defining the visibility v_{AB} of an object. Rose [3] found that objects can be reliably distinguished when v_{AB} is greater than 4 or 5.

$$v_{AB} = CNR\sqrt{n} \quad (2)$$

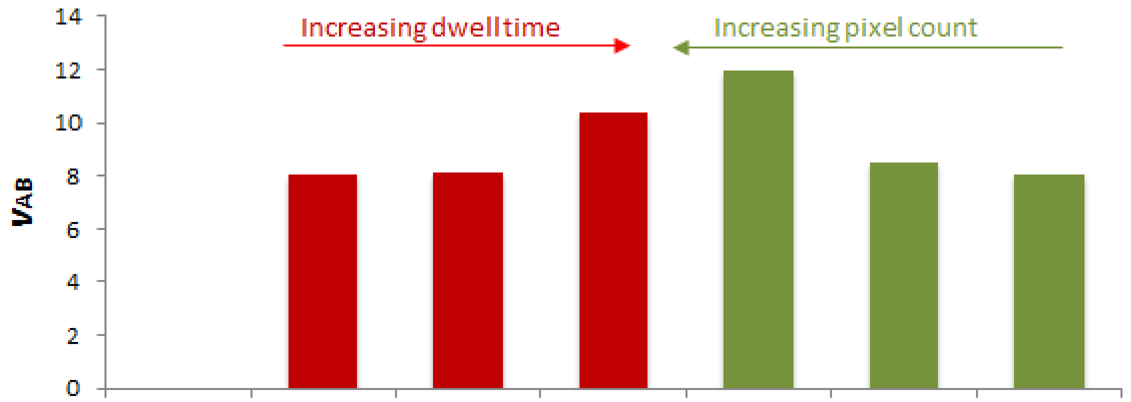

Dimension	512px	512px	512px	2048px	1024px	512px
Dwell Time	19.5μs	78μs	312μs	19.5μs	19.5μs	19.5μs
Acquisition Time	5.1s	20.4s	81.8s	81.8s	20.4s	5.1s

Acquisition Parameters

Fig. S1. Plot of the visibility v_{AB} of single atoms against different acquisition parameters. Single atoms and the C-film were sampled in a 0.3nm^2 region and the average grey scale value measured. The results of 30 repeats are then used to determine S_A, S_B and σ_0.

Blurring of single atoms is not observed in the range of scan speeds used, as the intensity profile typically measured is Gaussian. Thus the scan rate is not the same as the migration speed and at least a fraction of the single atoms present are being detected with a scan rate much greater than the migration speed.

Figure S1 shows the expected improvement in v_{AB} as the acquisition time is increased from 5.1s to 81.8s. Note that there is no associated decrease in single atom number for dwell time or pixel size variations. This implies that the scan speeds used in the 5.1-81.8s range are not lower than the typical migration speed.

The maximum dwell time used is 312μs, corresponding to 512x512 pixels, with a pixel size of 0.07nm. If the pixel size exceeds the probe size (>0.1nm), then there will be gaps in the raster scan, potentially leading to undetected single atoms. As previously discussed, the acquisition time is limited to 81.8μs to reduce thermal drift. Maximising pixel count (2048x2048, 19.5μs) provides a v_{AB} of 12, versus 10.4 when dwell time is increased to 312μs, 512x512 pixels. The difference between the two is detectable, but likely not significantly different when the temperature or support is altered. However, maximising pixel count does reduce the electron beam dose rate to the sample. Most studies suggest that the significant factor in reducing beam effects is a dependency on dose rate, rather than total dose [4-7]. The following work therefore uses 2048x2048 pixels, at a dwell time

of 19.5μs. Note that rastering of the e^- beam pauses at the beginning of each line, thus the acquisition times are slightly longer than those stated for comparison purposes above.

The final parameters of interest are the brightness and contrast levels used to interpret the detector signal. These are kept at constant values where contrast is maximised (for optimised differentiation between Pt and C) and brightness allows for visibility of internal particle structure, single atom resolution and some substrate visibility (brightness was approximately 1/3 of the maximum value). Optimised conditions for single atom detectability at 20˚C have now been developed and an understanding of in situ OR at the atomic scale can be undertaken.

Electron Beam Effects

Careful calibration experiments [8-11] were employed in the experiments. Minimally invasive low dose electron beam techniques were used throughout. In situ ESTEM data under low dose imaging conditions were compared to regions unexposed to the electron beam by beam blanking [11]. The aim was to ensure non-invasive characterisation. From Fig. S2. there is no change in either the PSD shape or the mean particle size of the areas exposed or unexposed to the electron beam. This indicates that electron beam influence has been minimised by using a sampling rate of 1 acquisition per 5 minutes.

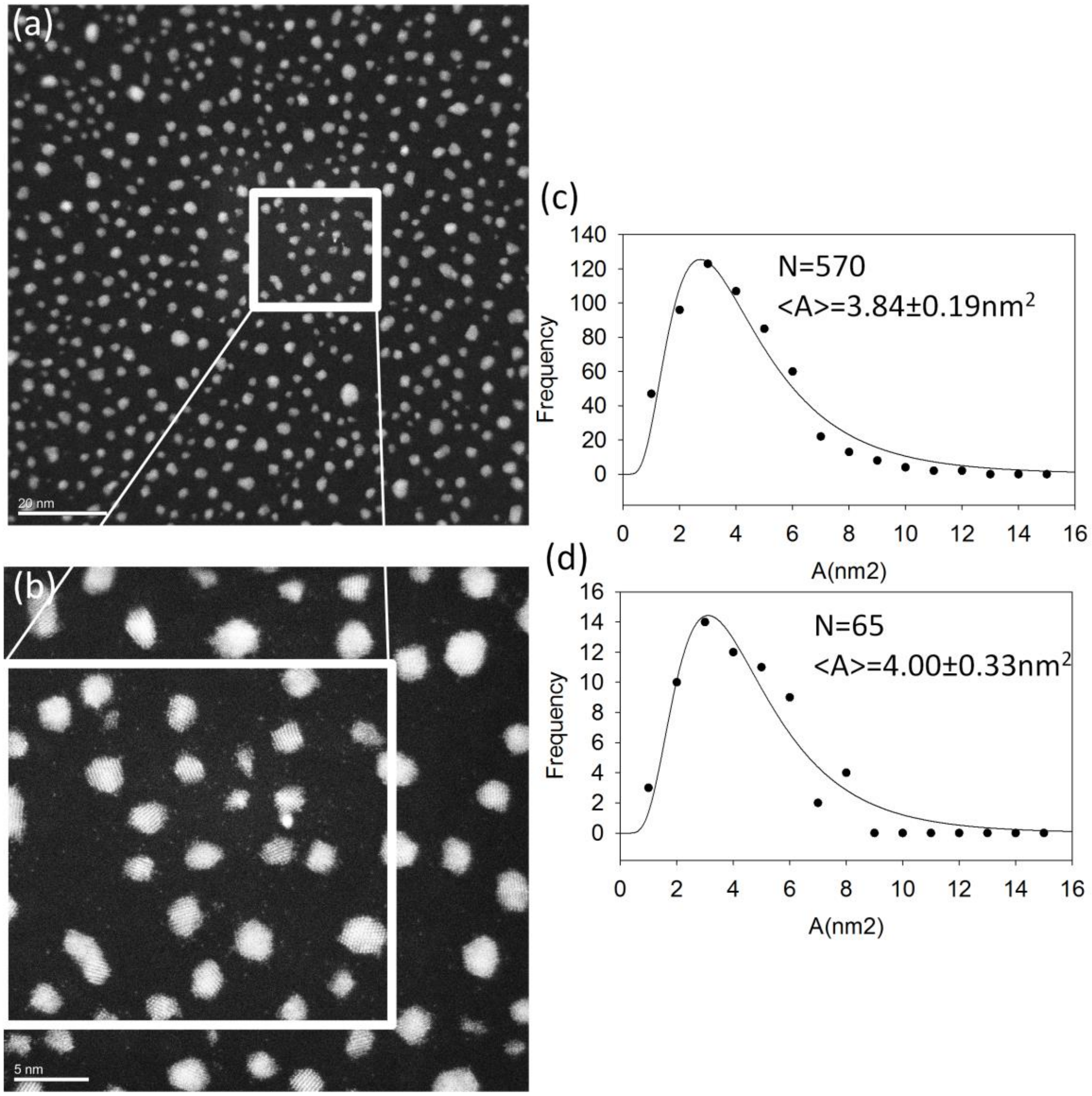

Fig. S2. Investigating beam effects by sampling the area surrounding the experimental region. (A) Low magnification (1.2MX) image of experimental area after 75 minutes at 250˚C, 3Pa H_2. (B) 4MX high magnification image of the 23.5x23.5nm experimental area after 75 minutes, (C)PSD with lognormal fits of low and high (D) magnification images, showing that the mean particle size agrees within error.

Over the course of the 75 minute experiment, 16 high magnification (4MX) images were taken. The positions of the single atoms and particles in each frame were calculated relative to 4 large

particles in the corners of each image. This minimises the influence of particle migration, although none was detected above the noise due to particle shape changes. The error in single atom position is thus the difference between alignment of the four particles in each image.

2. **Voronoi Averaging Effects**

The Voronoi analysis assumes that the local correlation model (using a Voronoi averaging technique) is applicable to this situation. To ensure that the single atom density changes are not an averaging effect, the observation area was changed to the individual Voronoi cells surrounding particles 17 and 18, Fig. S3. This shows a similar decrease in single atom density at t=25 mins, and increase at t=55 mins, showing that the trend is not an artefact introduced by surrounding particles and their nearest neighbours. The single atom density can also obviously be affected by focussing errors or other misalignments when acquiring images. If this was influencing the 'bathtub' trend from Fig. S4., then the single atom count across the image would follow the same trend. From Fig. S4., it can be seen that this is clearly not the case.

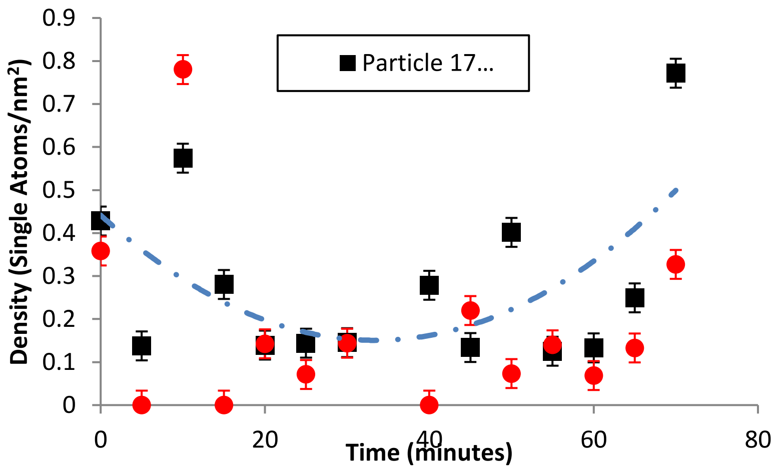

Fig. S3. Single Atom density in individual Voronoi cells of particle 17 and 18. A second order polynomial fit is shown by the dashed blue line. The initial decrease and post decay increase in single atom density is shown to be representative of particle 17 and 18 Voronoi cells.

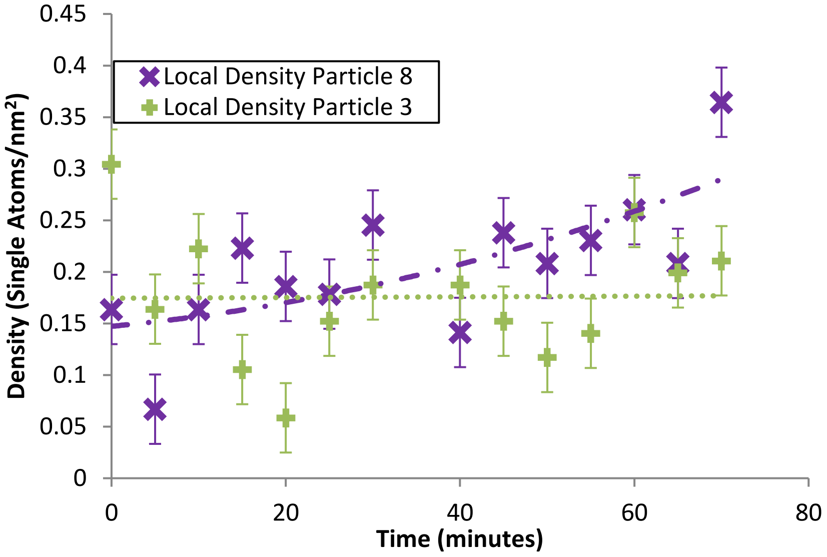

Fig. S4. The single atom density in the nearest neighbour regions of particles 3 and 8. Particles 3 and 8 are disordered and of similar size to particles 17 and 18. However, the local single atom density does not decrease during the experimental time frame, possibly due to the influence of nearest neighbour particles.

3. Acknowledgments

The authors acknowledge support from the UK Engineering and Physical Science Research Council (EPSRC) for a research Grant EP/J0118058/1 to P.L.G. and E.D.B., a PhD studentship to T.E.M. and a postdoctoral research assistantship to R.W.M. from the grant, in Boyes and Gai group.

4. References

1 Morgenstern, K. Fast scanning tunnelling microscopy as a tool to understand changes on metal surfaces: *physica status solidi (b)* **242**, 773-796 (2005).

2 Slater, J. C. Atomic radii in crystals. *The Journal of Chem Physics* **41**, 3199-3204 (1964).

3 Rose, A. The sensitivity performance of human eye. *JOSA* **38**, 196-208 (1948).

4 Batson, P. E. Motion of gold atoms on carbon in the aberration-corrected STEM. *Microscopy and Microanalysis* **14**, 89-97 (2008).

5 Jinschek, J. Advances in the environmental transmission EM for nanoscale reaction. *Chemical Communications* **50**, 2696-2706 (2014).

6 Helbeg, S. *et al.* Observing gas-catalyst dynamics at atomic resolution. *Micron* **68**, 176-185 (2015).

7 Woehl, T. J., Evans, J. E., Arslan, I., Ristenpart, W. D. & Browning, N. D. Direct in situ determination of the mechanisms controlling nanoparticle nucleation and growth. *ACS nano* **6**, 8599-8610 (2012).

8 Gai, P. L., Boyes, E. D., Hansen, P. L. Helveg, S., Giorgio, S. & Hentrey, C. Atomic-resolution environmental transmission electron microscopy for probing gas–solid reactions in heterogeneous catalysis. *MRS bulletin* **32**, 1044-1050 (2007).

9 Gai, P. L. & Kourtakis, K. Solid-state defect mechanism in vanadyl pyrophosphate catalysts: Implications for selective. *Science* **267**, 661-663 (1995).

10 Boyes, E. & Gai, P. Environmental high resolution electron microscopy and applications to chemical science. *Ultramicroscopy* **67**, 219-232 (1997).

11. Boyes, E.D., LaGrow, A.P., Ward, M.R., Mitchell, R.W. & Gai, P.L. *Acc.Chem.Res*. ***53***, 390 (2020).
